# False beliefs can bootstrap cooperative communities through social norms

**DOI:** 10.1017/ehs.2021.30

**Published:** 2021-06-14

**Authors:** Bryce Morsky, Erol Akçay

**Affiliations:** University of Pennsylvania, Philadelphia, PA, USA

**Keywords:** Community emergence, conditional cooperation, expectations, social norms

## Abstract

Building cooperative communities is a crucial problem for human societies. Much research suggests that cooperation is facilitated by knowing who the cooperators and defectors are, and being able to respond accordingly. As such, anonymous games are thought to hinder cooperation. Here, we show that this conclusion is altered dramatically in the presence of conditional cooperation norms and heterogeneous beliefs about others’ behaviours. Specifically, we show that inaccurate beliefs about other players’ behaviours can foster and stabilise cooperation via social norms. To show this, we combine a community's population dynamics with the behavioural dynamics of their members. In our model, individuals can join a community based on beliefs generated by public signals regarding the level of cooperation within, and decide to cooperate or not depending on these beliefs. These signals may overstate how much cooperation there really is. We show that even if individuals eventually learn the true level of cooperation, the initially false beliefs can trigger a dynamic that sustains high levels of cooperation. We also characterise how the rates of joining, leaving and learning in the community affect the cooperation level and community size simultaneously. Our results illustrate how false beliefs and social norms can help build cooperative communities.

## Graphical abstract

**Figure:**
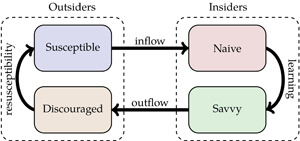

**Social media summary:** In a model, we show how distorting beliefs can get cooperative communities started and sustain them.

## 1. Introduction

The ecological and evolutionary success of our species is in large part dependent on our ability to start and grow cooperative communities where individuals work together for common aims beyond their immediate kin. Humans are exceptionally good at cooperating with one another, and understanding the underpinnings of such cooperation has been a primary goal of evolutionary human sciences for decades. Extensive experimental evidence shows that human cooperation (and any other social behaviour) is contingent and is affected by others’ behaviours (Chaudhuri, 2011; Fischbacher, Gächter, & Fehr, 2001; Frey & Meier, 2004) and social norms about own and others’ behaviours (Bicchieri & Chavez, 2010; Bicchieri, Dimant, Gächter, & Nosenzo, 2020; Kimbrough & Vostroknutov, 2016).

Social norms can be defined as the rules of the game in social interactions (Bicchieri, 2006). Many norms prescribe what norms holders should do given what others do or what situations individuals encounter. The content of these norms evolves culturally (Gelfand & Jackson, 2016), yet humans have an innate predisposition for internalising culturally acquired norms (Gintis, 2003). Such social norms can instigate group-beneficial behaviours (Akçay & Van Cleve, 2021; Gavrilets & Richerson, 2017; Gintis, 2003) or coordinate social behaviours for mutual gain (Gintis, 2010; Morsky & Akçay, 2019). Importantly, adopting a social norm does not mean that individuals blindly follow them: rather, social norms frequently act to shape preferences or default behaviours of individuals, who still make decisions that are responsive to their payoffs or other signals and beliefs. For example, an important social norm is conditional cooperation (Bicchieri, 2010; Bicchieri, Dimant, & Sonderegger, 2020b), wherein norm holders will cooperate if they believe that others are cooperating. This kind of conditional behaviour can be generated by individuals having ‘relational utilities’, i.e. utility of non-material ‘goods’ arising from social interactions such as the enjoyment gained by watching a movie with friends (Traxler & Spichtig, 2011). Relational utility earned from cooperating can make cooperation more rewarding (or non-cooperation more aversive) if individuals know or expect others to cooperate. If the proportion of such norm holders is high, then this reciprocity norm can sustain cooperation. Such conditionally cooperative preferences can account for the emergence of cooperative clusters in social networks even if partner choice for cooperators is weak (Ehlert, Kindschi, Algesheimer, & Rauhut, 2020).

Yet, building a cooperative community through conditionally cooperative norms faces an obvious problem: if others are not cooperating, conditional cooperators will not either. Furthermore, a robust finding from experimental games is that even if one starts out with substantial amounts of cooperation, the presence of selfish individuals or imperfectly conditional cooperators almost invariably unravels cooperation over time (Fischbacher & Gachter, 2010; Fischbacher et al., 2001; Thöni & Volk, 2018). Conditional cooperation norms therefore cannot by themselves get cooperation off the ground, and are subject to erosion even when starting from high levels of cooperation.

A related problem is that if a community is not cooperative, there will be little incentive to join it and strong incentives to leave it, such that communities that cannot maintain cooperation might cease to exist altogether. Thus, concurrently with the problem of maintaining cooperation among their members, communities also face the problem of recruiting and retaining members.

Human societies had to solve these problems over and over again in history to build and maintain cooperative communities. They are still extremely salient in contemporary life, including in online communities, which represent a rich substrate to study how cooperative communities emerge and are maintained. One example that has been studied in some detail is file-sharing software that relies on users’ willingness to provide uploads of files for others. These communities have instituted a variety of rules to mitigate free-riding (downloading data without uploading; Harris, 2018), and previous models have explored the roles of reputation (Lai, Feldman, Stoica, & Chuang, 2003), micropayments (Golle, Leyton-Brown, Mironov, & Lillibridge, 2001) and reducing congestion (Krishnan, Smith, Tang, & Telang, 2004) on facilitating cooperation.

Strahilevitz (2003) termed technology that exploits social mechanisms to induce cooperation amongst its users ‘charismatic code’. Charismatic code can work by distorting participants’ normative expectations by masking cheating, exaggerating levels of cooperation and promoting collective identity and prosocial norms. For example, the file-sharing software Morpheus would depict the total number of users and files being shared, which suggested a very high share rate per person (Strahilevitz, 2003). However, this hid the underlying heterogeneity in sharing where the majority of users shared relatively little. This omitted truth portrayed a cooperative community in which everyone gave back what they took. This in turn fuelled internal norms of reciprocity and conditional cooperation, which have been found among file sharers (Cenite, Wanzheng Wang, Peiwen, & Shimin Chan, 2009), thereby stimulating more cooperation than there otherwise would be. Charismatic code therefore provides an explanation for why there can be substantial trust and cooperation in anonymous groups of file sharers.

This phenomenon can be seen in other contexts as well (Strahilevitz, 2003). An example is binge drinking norms (Lewis & Neighbors, 2006; Perkins, 1997), which can be fostered by conspicuous signalling and conditional following of the norm (here the norm is to binge drink). Students often overestimate the level of binge drinking and thus drink more than they otherwise would. Those who are heavily intoxicated are likely to be more visible than those not, which contributes to this. Thus, these beliefs can become self-fulfilling prophecies. Understanding this, campus programmes that publicise the lower than expected levels of binge drinking have lowered its prevalence (Haines, 1996). In a similar way, publicising compliance can increase compliance via conditional cooperation with respect to tax compliance (Coleman, 1996; Traxler, 2010). The interplay between expectations and norms also has implications for vaccination decision making (Xia & Liu, 2014). These examples suggest that distorted or hidden information can affect the success of cooperation and other social norms in communities through changing individuals’ beliefs. Yet the joint dynamics of beliefs, behaviours and community formation in the face of distorted information remain relatively unexplored.

Our goal in this paper is to understand how distortions in the signals individuals are getting about others’ behaviours can affect the dynamics of conditional cooperation norms. Specifically, we are interested in when such distortions can get cooperative communities started and maintain them. To explore these questions, we build a mathematical model of the growth and decline of a community as individuals join and leave it, as well as the joint dynamics of individual behaviour and beliefs as a consequence of (potentially distorted) signals and individual learning.

We model individuals within the community that earn material utility from a public goods game, as well as experiencing ‘relational utility’ which represents internalised conditional cooperation norms (Akçay & Van Cleve, 2021; Gavrilets & Richerson, 2017; Traxler & Spichtig, 2011). Specifically, we assume that individuals experience utility (‘warm glow’) or disutility (‘guilt’) based on their own cooperation level relative to their *perception* of the mean cooperation level in the community. Depending on the strength of this relational utility, each individual has a threshold perceived level of cooperation at which they too cooperate. Individuals within the community can learn the true level of cooperation through social learning (Rendell et al., 2010), which is ubiquitous in human behaviour and a salient factor when cooperative environments foster further cooperation (Ehlert et al., 2020). Those outside of the community also have a perception of the level of cooperation within the community, which may be a high naive amount or a low amount (for those who were previous members but now discouraged). Outsiders that view the community positively may then enter it with that belief determining the level of cooperation they will engage in, while insiders who have learned the true level of cooperation can become discouraged and leave the community. We show that the relative rates of entering communities, learning the true level of cooperation and leaving determine whether a cooperative community can exist and how much cooperation can be sustained.

## 2. Model

### 2.1. Basic conditional cooperation model

We begin with a model of a public goods game with a norm of conditional cooperation from Traxler and Spichtig (2011), in which individuals cooperate (donate to a public good) if a sufficient proportion of the community cooperates. Individuals have a choice of whether to donate to a public good or not, and they make it by maximising their utility, which is a combination of components both material, the public good and cost to donate, and relational, the ‘warm glow’ from obeying or guilt from disobeying the norm. In the case of the file-sharing example, material utility is derived from files downloaded while relational utility comes from the feeling of giving back to the community. Calculating the maximum utility leads to thresholds at which individuals will donate. These thresholds will depend upon the proportion of others who donate as well as an individual's own sensitivity to the norm. With the norm varying within the population, this framework produces a threshold effect akin to the classic Granovetter–Schelling model, where individuals are assumed to change their behaviour once the population average behaviour crosses some threshold. Schelling used this framework to study segregation (Schelling, 1969, 1971). Granovetter and Soong further developed threshold models to understand the emergence of riots, innovation, rumours, voting, migration, strikes and consumer demand (Granovetter, 1978; Granovetter & Soong, 1983, 1986, 1988).

In our model, we define *F*(*p*) to be the cumulative density function of the norm sensitivity, i.e. *F*(*p*) is the proportion of the population that will cooperate given that proportion *p* of the community is cooperating (we use *f*(*p*) = *F*^′^(*p*) for the probability density function). This function may be derived from utility maximisation as in Traxler and Spichtig (2011) (see Supporting Information-1 for details) or simply assumed. Individuals will change their behaviour relative to the difference between their normative value and the level of cooperation. Thus, assuming that individuals can observe the true level of cooperation, the dynamics are determined by the equation
1
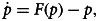

which is a continuous time version of the Granovetter–Schelling model of threshold behaviour (Granovetter, 1978; Granovetter & Soong, 1983, 1986, 1988; Schelling, 1969, 1971). This dynamic can be interpreted on different time scales. With respect to the file-sharing example, learning occurs for an individual over a period of their life. However, we can also interpret this dynamic as cultural evolution where this learning dynamic occurs over longer time scales (Henrich & McElreath, 2003).

**Figure 1.**
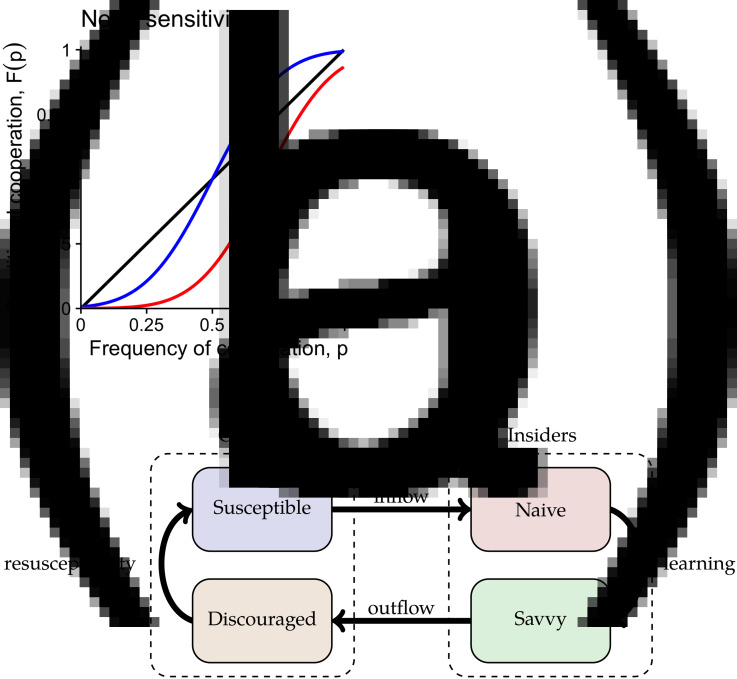
(a) The frequency of cooperation given that a proportion *p* are cooperating for two different distributions of norm sensitivity. Here, *F*(*p*) is the cumulative distribution function of a normal distribution with means *μ* = 0.5 and 0.7 for the coordination (blue curve) and cooperation (red curve) dilemmas, respectively; the variance is *σ*^2^ = 0.04 for both. For the coordination dilemma, we have a bistable system with stable states of high cooperation and low cooperation. For the cooperation dilemma, we have a sole stable fixed point with low cooperation. Note here that there is no deception or misinformation; players know the true level of cooperation. (b) Diagram of the four-compartment model. Susceptible individuals enter the community as new naive insiders. Learning through community interactions, they may become savvy insiders who know the true level of cooperation. If there is a discrepancy between the true level of cooperation and the naive expectations, savvy insiders may become discouraged and leave the community. Discouraged players then can become susceptible (again).

The dynamics in eqn (1) reach equilibrium when 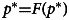, which can happen at multiple points. At least one such equilibrium exists and is stable (which for parameters we are concerned with will be for low cooperation). In some parameter regimes, we can have three equilibria: one stable low cooperation, one unstable intermediate cooperation and one stable high-cooperation equilibrium, relatively speaking. Figure 1a depicts both the three and one equilibria cases, which we will denote as the coordination dilemma (blue curve) and the cooperation dilemma (red curve), respectively. The coordination dilemma is thus a bistable system, where the population is stable when either most cooperate or few do. The cooperation dilemma is stable only at low levels of cooperation. The type of dilemma switches between these depending on the parameters of the *F*(*p*) curve. This model is our base setting. Although it is framed in terms of cooperation, it applies more generally to conditional adherence to a behaviour. Below, we show that distorting the beliefs about the behaviour within the community can alter these equilibria to maintain a stable community with high levels of cooperation.

### 2.2. Model of community and belief dynamics

Our goal is to ask how unfounded and potentially inaccurate beliefs might affect the social dynamics in a community. To do that, we extend the model above to include agents who (perhaps mistakenly) believe that the community is highly cooperative when they enter it. These unfounded beliefs might be due to optimism bias (McKay & Dennett, 2009; Sharot, 2011), deliberate misrepresentation and obscuring, or to salience biases (see Discussion for more on this). After joining the community, however, individuals can learn about the true level of cooperation, and if disillusioned, leave. To represent these dynamics, we use a compartmental model (as depicted in Figure 1b) with four classes of individuals: two currently belonging to the community in which the public goods game is played (insiders), and two that do not belong to it (outsiders). Individuals can transition between these classes as depicted in Figure 1b.

The community recruits new members from outsiders that are susceptible. Specifically, susceptibles enter the community as new naive members at a rate proportional to their contact rate with community members: 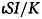 where 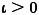 is the inflow rate and *K* is the total population size. This captures the intuition that susceptible outsiders will become aware of the community (and joining opportunities) by meeting community members, or being exposed to them, both of which are more likely the bigger the community is. Insiders in turn belong to two classes: ‘naive’ and ‘savvy’ (fractions 1 − *y* and *y*, respectively). All newly joined individuals are initially naive, and believe that the level of cooperation in the community is 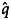, regardless of the true level of cooperation. Thus, a fraction 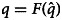 of naive individuals will cooperate initially.

However, the naivety cannot last forever: we assume that naive insiders learn the true level of cooperation from their own interactions or observations of others. When this happens, naive individuals turn into savvy individuals. Depending on what the true level of cooperation is, savvy individuals might cooperate less than naive ones. We assume that the rate at which naive individuals learn is proportional to how different the naive beliefs are from reality. In particular, we assume that naive insiders become savvy at per capita rate 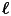 where 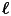 is the parameter that modulates learning speed, 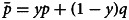 is the mean (actual) level of cooperation among insiders, *p* is the frequency of cooperation among savvy insiders and *y* is the proportion of insiders that are savvy. This equation captures the intuition that big differences between expectations of naive individuals and reality will be apparent faster than small differences. Note that *p* and *y* (and therefore 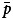) are state variables that depend endogenously on the community dynamics.

Once they become savvy, individuals might become disillusioned and leave the community. We assume that leaving occurs at a rate proportional to the difference between their expectations and reality, 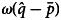, where *ω* is an outflow rate parameter. This again captures the intuition that a bigger gap between initial expectations and reality would create stronger disillusionment and a more powerful motivation to leave the community. Note that we assume that naive beliefs are higher than the true level of cooperation, as our main concern is to evaluate if rosy beliefs might bootstrap cooperation. In the opposite case where naive beliefs are lower than the true level, the maintenance of cooperation would be more difficult, although as savvy individuals (being pleasantly surprised) would actually increase their cooperation level and never leave, the influx of naively pessimistic newcomers could drive down cooperation.

Savvy players that have left the community become discouraged outsiders and are not directly susceptible to reentry. However, we assume that their discouragement can wane, and thus discouraged individuals might become susceptible again at a rate φ, reflecting the waning of the memory of their experience in the community.

The following equations describe the dynamics explained above, using four state variables: the number of susceptibles (*S*) and insiders (*I*), the proportion of insiders that are savvy (*y*) and the frequency of cooperation among savvy insiders (*p*) (see Table 1 for a list and description of variables and symbols; Supporting Information-2.1 gives the full derivation of these equations from the system in Figure 1b.):
2
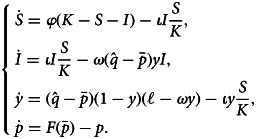

**Table 1.** Summary definitions of parameters and variables

Parameter/variable	Description
*μ*	Mean norm sensitivity
*σ*^2^	Variance in norm sensitivity
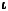	Inflow rate
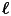	Learning rate
*ω*	Outflow rate
φ	Resusceptiblity rate
*I*	The number of insiders
*K*	The total number of individuals
*S*	The number of susceptible individuals
*y*	The proportion of insiders that are savvy
*p*	The frequency of cooperation among savvy insiders
*q*	The frequency of cooperation among naive insiders
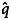	The naive insiders’ belief of the frequency of cooperation

Throughout, we consider both the coordination and cooperation dilemmas. Although our analytical results apply to any distribution of thresholds *F* of the shapes given in Figure 1a, for numerical results and graphing purposes, we assume that *F* is the cumulative distribution function of a normal distribution with means *μ* = 0.5 and 0.7 for the coordination and cooperation dilemmas, respectively, and, unless otherwise stated, variance *σ*^2^ = 0.04. Note that the normal distribution is defined over (−∞, + ∞), whereas we are restricted to the domain [0, 1]. Thus, *F*(0) > 0 and *F*(1) < 1 mean that some fraction of individuals will cooperate even if no others do and some fraction will never cooperate even if all others do. We also assume for our plots that the naive belief is the high-cooperation solution to eqn (1) in the coordination dilemma with the above parameters (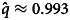). We determine the joint equilibria for insider and outsider populations, their compositions and the cooperation level amongst the insiders. In doing so we explore the parameters, as summarised in Table 1, to see how they impact the qualitative behaviour of the model.

## 3. Results

### 3.1. Coordination dilemma

We first consider a coordination dilemma, which is characterised by three equilibria in the basic model without community dynamics. In the community dynamics model, the high-cooperation equilibrium is always a stable equilibrium where all players are in the community and are cooperating at the same high level (see Supporting Information-2.2, Theorem 1).

Whether or not we have more equilibria depends on two ratios: the ratio of outflow to learning rates, *ω*/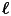, and the ratio of the inflow rate to the rate at which insiders become discouraged,
3
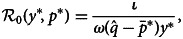

evaluated at equilibrium (see Supporting Information-2.2). To take up the latter first, 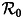 effectively measures whether the community will grow from zero, analogous to the concept of the basic reproduction number in epidemiology. Accordingly, a mixed equilibrium, where some of the population is in the community and some outside, exists only if 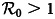. This mixed equilibrium of insiders and outsiders as functions of 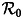 is:
4
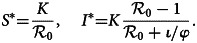


If 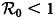, the only stable equilibrium is one where everyone is an outsider, capturing the intuition that for the insider population to be positive at equilibrium, its growth rate from zero has to be positive.

**Figure 2.**
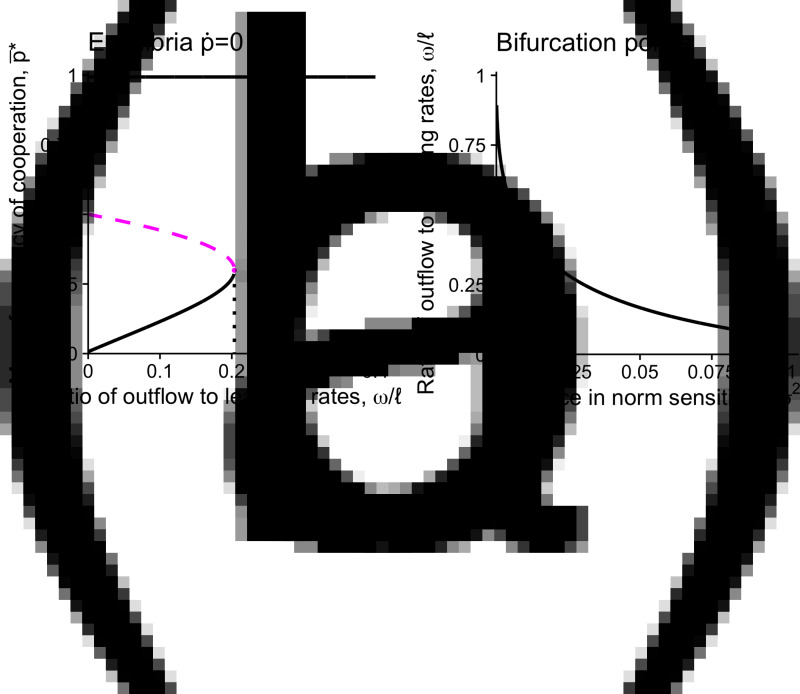
(a) The solid black and dashed magenta curves represent the stable and unstable equilibria, respectively, while the dotted line marks a qualitative change in the system. Increasing the ratio of outflow to learning rates annihilates the lower and medium equilibria leaving only the high-cooperation equilibrium. (b) The bifurcation point at which this shift occurs is decreasing for increasing variance in the normsensitivity.

The ratio of outflow to learning rates, *ω*/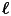, affects the existence and stability of additional equilibria through changing the fraction of savvy individuals *y* and the mean cooperation level 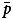. At a low ratio of outflow to learning rates, there may be two equilibria in addition to the high cooperation one: one stable with low cooperation and another unstable with moderate cooperation. These equilibria both solve 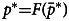; in other words, they are obtained when the savvy individuals’ cooperation rate equals the cumulative distribution of norm sensitivities at the true (averaged between savvy and naive individuals) cooperation rate (see Supporting Information-2.2 for the stability conditions for these equilibria). Both of these equilibria will have a mixture of insiders and outsiders, given by eqn (4). As the ratio of outflow to learning rates increases, these two solutions converge until they annihilate each other, leaving only the high-cooperation equilibrium described before. Thus, by decreasing the rate of learning by naive individuals or increasing the rate at which savvy individuals leave, a low-cooperation community can shift to become a high-cooperation community (Figure 2).

**Figure 3.**
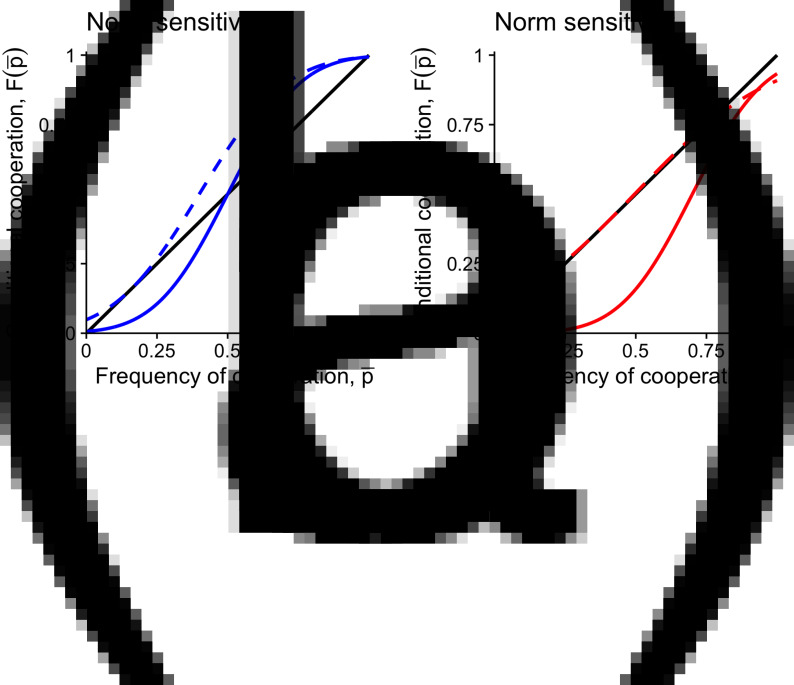
The presence of naive individuals shifts the norm sensitivity of savvy individuals, *F* (solid coloured), up to the dashed curves. This shift occurs in both the coordination (a) and cooperation (b) dilemmas.

How this transition happens can be seen by first considering the proportion of savvy insiders at equilibrium. Provided that both the insider and outsider communities are non-zero at equilibrium, the equilibrium proportion of savvy insiders is *y*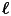 = 1/(1 + *ω*/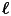). The proportion of savvy insiders will decrease as the ratio of outflow to learning rates increases, since savvy individuals leave faster than naive individuals become savvy. Because naive individuals cooperate at higher levels than savvy individuals, this increases the average level of cooperation in the community, in turn inducing more savvy individuals to cooperate as well. This means that the blue curve 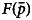 from Figure 1a is shifted up, as depicted in Figure 3a. The higher the ratio of outflow to learning rates is, the lower the fraction of savvy individuals at equilibrium, and the more the curve will be shifted upwards. For a sufficiently large shift upwards, all but the high equilibrium can be annihilated. Figure 2a shows that as the ratio of the outflow to learning rate increases, the intermediate equilibrium of 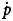 decreases while the low-cooperation equilibrium increases. These two equilibria meet and annihilate one another at a bifurcation point, a point where the system's behaviour qualitatively changes. This happens when the norm sensitivity curve becomes tangent to the diagonal at the equilibrium cooperation level of the savvy insiders, given by 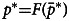. For outflow to learning rate ratios higher than this point, the only equilibrium is high cooperation.

Interestingly, the threshold ratio of outflow to learning rate where the low-cooperation equilibrium disappears decreases as the variance in norm sensitivity increases, as Figure 2b shows. This means that as more individuals become unconditional (or almost unconditional) cooperators and defectors (with high and low norm sensitivities), the high cooperation-only state becomes easier to reach. The intuition behind this is that, as the variance increases, the lowest equilibrium *p*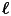 increases towards the middle equilibrium, as more individuals will be close to unconditional cooperators. As such, the solid blue curve in Figure 3a needs to be shifted up less for the low-cooperation equilibrium to be destroyed. This makes highly cooperative communities globally stable at higher learning (or lower outflow) rates.

**Figure 4.**
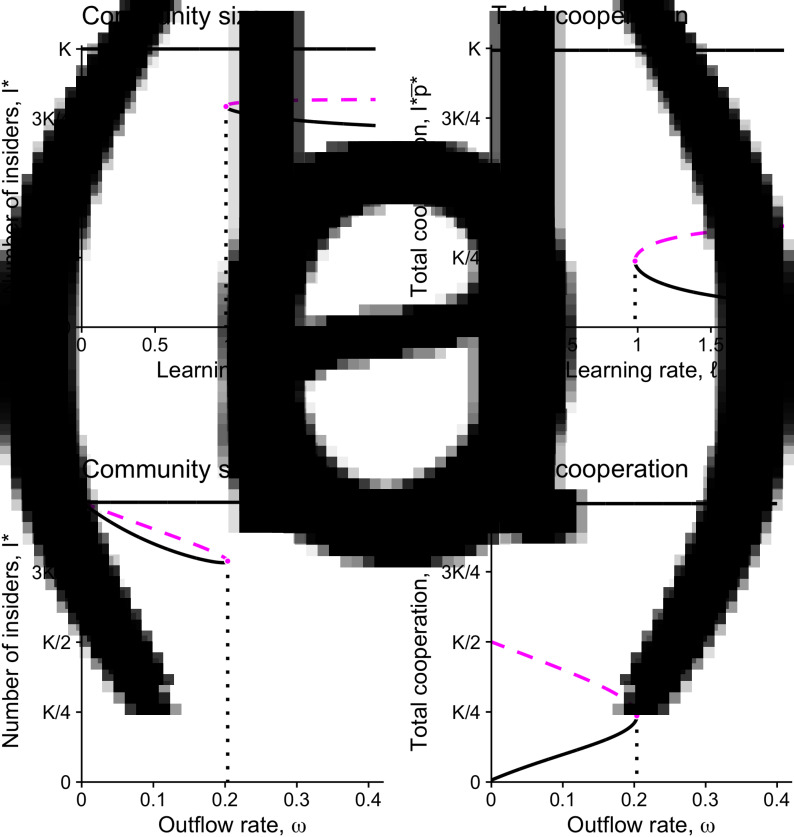
(a,b) Increasing the learning rate creates two new equilibria. Here, 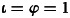 and *ω* = 0.2. (c,d) Increasing the outflow rate will reduce the community size yet increase the total amount of cooperation. For sufficiently high outflow rate, the low and middle equilibria are destroyed. Here, 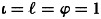. The solid black and dashed magenta curves represent the stable and unstable equilibria, respectively, while the dotted lines mark qualitative changes in the system.

Next, we consider the impact of the parameters on the community size, i.e. the total number of insiders, *I*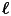, and total amount of cooperation across all individuals. The number of insiders increases with respect to increasing inflow and resusceptibility rates. Further, since the equilibrium values *y*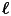 and *p*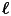 are not affected by these parameters, increasing the number of insiders will increase the total amount of cooperation in the population. The impact of learning and outflow rates are more complicated. Figure 4a and b shows that for low learning rates, there is no equilibrium with both insiders and outsiders; the entire population will be in the community. However, sufficiently increasing the learning rate produces two new equilibria: one unstable and one stable with a relatively lower community size and total level of cooperation. Figure 4c and d depicts the results for varying outflow rates. For low outflow rates, there are three equilibria, and for high outflow rates there is only one. Up to the point where this change occurs, increasing the outflow rate reduces the community size of both smaller community size equilibria. However, it increases the total level of cooperation for the stable equilibrium and decreases it for the unstable one.

### 3.2. Cooperation dilemma

We next turn to the cooperation dilemma, which is characterised by a norm sensitivity curve that mostly lies below the diagonal, like the red curve in Figure 1a. This means that a community of all savvy individuals would have low cooperation, and even naive individuals that come in with a high estimate of frequency of cooperation themselves will not cooperate at that high level. Nonetheless, our results show that depending on the ratio of the outflow to learning rates, a highly cooperative equilibrium community can be achieved.

**Figure 5.**
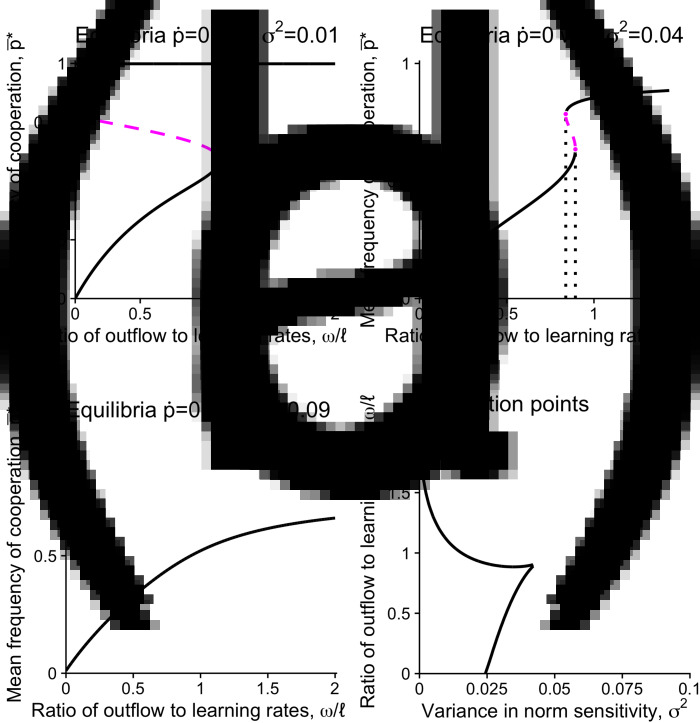
(a) For low variance, *σ*^2^, we have a coordination dilemma. (b) For higher variance, increasing *ω*/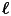 increases the equilibrium value of cooperation. For intermediate values, three equilibria are present. (c) With sufficiently high variance, there is only one equilibrium. (d) For low *σ*^2^, the system is a coordination dilemma as in (a), and thus the bifurcation point is decreasing for increasing variance in the norm sensitivity. As we increase *σ*^2^, we have two bifurcation points as in (b). For higher variance, we have no bifurcations and 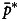 is increasing as in (c). For (a)–(c), the solid black and dashed magenta curves represent the stable and unstable equilibria, respectively, while the dotted lines mark qualitative changes in the system.

The only possible equilibria in the cooperation dilemma are one where everyone is an outsider (when 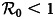) and the one where there is a mixture of insiders and outsiders given by eqns (4). For the latter case, Figure 5a–c shows how the cooperation level within the community changes with the ratio of the outflow to learning rates, and how this behaviour is modulated qualitatively by the variance in norm sensitivity. For low variance (Figure 5a), the system is in fact a coordination dilemma, with the same patterns as in the previous section. For higher variance (Figure 5b), we have a genuine cooperation dilemma, and for low ratios of outflow to learning rate, there is only a single equilibrium with low cooperation. The frequency of cooperation increases with the ratio of the outflow to learning rates, until two more equilibria are created. One of these is high-cooperation equilibrium and stable, and the other intermediate cooperation and unstable. Further increasing the ratio of outflow to learning rates causes the latter equilibrium to collide with and annihilate the low-cooperation equilibrium, leaving us with only one stable, high-cooperation equilibrium. However, this equilibrium still falls short of the aspirational cooperation rate, 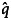, expected by naive individuals, because even naive individuals are not willing to cooperate at that level. For yet higher variance a single stable equilibrium exists throughout where the frequency of cooperation increases smoothly with the ratio of outflow to learning rates (Figure 5c). The aspirational cooperation rate 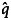 is not sustainable nor is there a discrete jump in cooperation. Yet we can increase cooperation at equilibrium as we increase the outflow rate relative to the learning rate, simply through increasing the proportion of naive individuals.

Figure 5d summarises the dynamical regimes we can observe. For low variance in norm sensitivity, we observe a coordination dilemma. The bifurcation point, i.e. the ratio of outflow to learning rates at which we switch from three equilibria to only one high cooperation one, decreases as the variance increases, as in Figure 2b. However, at *σ*^2^ ≈ 0.025 for these parameter values, the cooperation dilemma emerges, which features either one or three equilibria. The difference between the two curves is the region in which there are three equilibria. Above the higher curve, we have a sole relatively high-cooperation equilibrium. Below the lower curve, we have a sole relatively low-cooperation one. For sufficiently large variance, the bifurcation points disappear and the equilibrium level of cooperation simply monotonically increases as the ratio of outflow to learning rates increases. As in the coordination dilemma, cooperation is boosted by the presence of inaccurate beliefs about the level of cooperation, shifting the norm sensitivity (reaction curve) up as depicted in Figure 3b.

**Figure 6.**
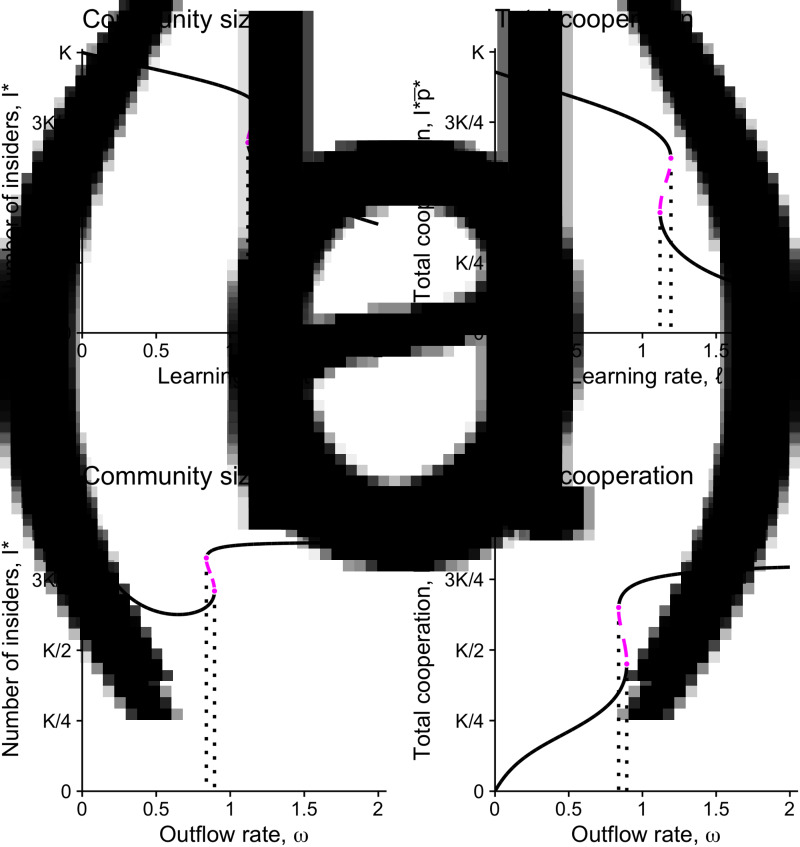
(a,b) Increasing the learning rate decreases the size of the community and the total amount of cooperation. Here the parameters are 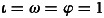. (c) Increasing the outflow rate will initially reduce the equilibrium number of insiders, after which it increases and eventually plateaus. (d) Increasing the outflow rate increases the total amount of cooperation in the population. For (c) and (d), 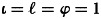. The solid black and dashed magenta curves represent the stable and unstable equilibria, respectively, while the dotted lines mark qualitative changes in the system.

Figure 6 shows the impact of the learning and outflow rates on the community size and total population. Increasing the learning rate generally reduces both the community size and total cooperation, as it reduces the fraction of naive individuals and the total rate at which individuals leave the community. Note that three equilibria can be created and then destroyed as the learning rate is increased (Figure 6a and b). Unsurprisingly, increasing the outflow rate from near zero will initially decrease the community size. More surprisingly, however, increasing the outflow rate further, the community size will start to increase again. This is because, as the fraction of savvy individuals declines mean cooperation levels go up and the actual leaving rate of savvy individuals (dependent on the difference between naive expectations and reality) will decline. Increasing the outflow rate further first creates a higher community size (and cooperation) and intermediate community size equilibrium, the latter of which again annihilates the lower community size equilibrium. After this point the community size plateaus, even though the total amount of cooperation in the population keeps increasing with the outflow rate.

### 3.3. Community crash and cycling

**Figure 7.**
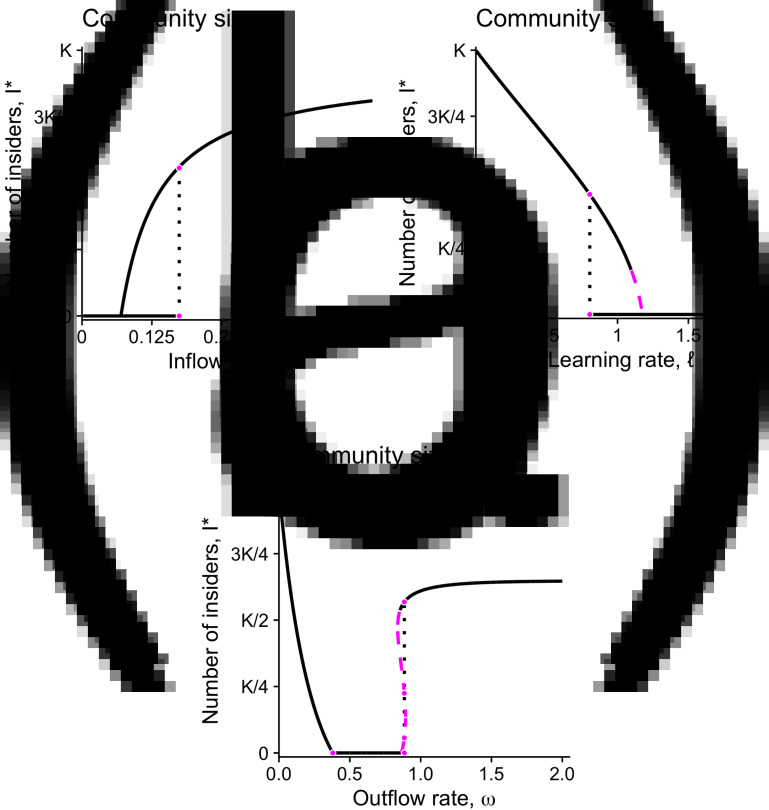
The parameters 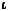, 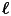 and *ω* determine whether or not the community may crash. (a) Low and high inflow rates always lead to a crashing or stable community, respectively, while intermediate rates lead to bistability. Here the parameters are 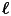 = *ω* = φ = 1. (b) Slow and fast learning always leads to a stable or crashing community, respectively, while intermediate rates lead to bistability. Here the parameters are 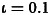 and *ω* = φ = 1. (c) There is a window in which the community can crash. Outside of this window, it cannot. Here the parameters are 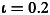 and 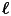 = φ = 1. The solid black and dashed magenta curves represent the stable and unstable equilibria, respectively, while the dotted lines mark qualitative changes in the system.

We next consider the conditions under which the cooperative community crashes in the cooperation dilemma. A community crash is when the number of insiders goes to zero and does not rebound. This occurs if 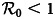 and 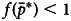 at this state (see Supporting Information-2.2, Theorem 2). Figure 7 plots the effects of different parameters on the existence of the crashing state. For low enough inflow rate, the crashing equilibrium is stable (equivalently, 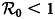; Figure 7a). However, as the inflow rate increases, a positive community size can also be stable in conjunction with the crashing equilibrium. Above a threshold inflow rate, the crashing equilibrium disappears and only the equilibrium with positive community size is stable. This pattern highlights the potential for path dependence (hysteresis) in the development of a community. For instance, consider a new community with an initially high inflow rate, e.g. because of its novelty or active recruitment efforts by the founders. If this initial inflow rate is sufficiently high, then the community can become established, and it can survive (albeit at smaller size) even if the inflow rate is reduced over time to a level that would have been insufficient to get the community off the ground.

The learning rate has the opposite threshold effect. For the crashing equilibrium to exist, the learning rate needs to be high enough (Figure 7b), and above this threshold there is again a range of learning rates where both the crashing and non-crashing equilibria are stable. Thus, the learning rate also exhibits potential for hysteresis, although in the opposite direction from the inflow rate. If the learning rate can be initially sufficiently suppressed (e.g. by making interactions within the community opaque), the community can emerge and stabilise. Once such a community is established the community will be robust against an increasing learning rate (up to a point).

Finally, the effect of the outflow rate differs from the inflow and learning rates in that it is non-monotonic (Figure 7c). For a low outflow rate, the community size is positive but decreasing as the outflow rate increases until the crashing equilibrium is the only stable equilibrium. This is intuitive: as individuals leave the community faster, it gets smaller and may not be able to sustain itself. Less intuitive is the fact that there is another threshold outflow rate above which the crashing equilibrium is unstable again, and a positive community size is stable. This happens because the high potential leaving rate of savvy individuals initially leaves the community mostly composed of naive ones and high cooperation, which reduces the realised leaving rate of savvy individuals. This allows the cooperative community to be stable again. The coordination dilemma exhibits similar threshold behaviour at which the community can crash (see Supporting Information-3). The regions of parameter space where crashing occur are depicted qualitatively in Supporting Information-2.2.

**Figure 8.**
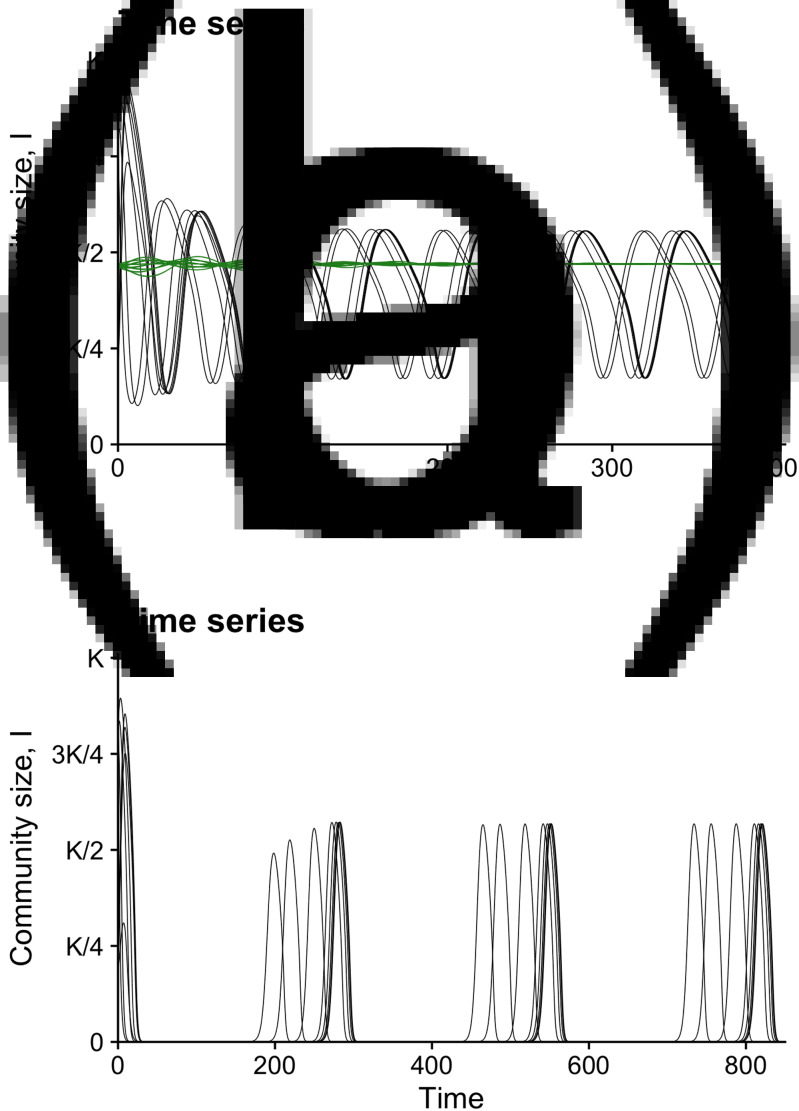
We observe cycles for the cooperation dilemma. (a) The time series for inflow and resusceptibility rates 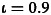 and φ = 0.09. Time series for initial conditions within 1.5% of the stable equilibrium are plotted in green. Other trajectories that lead to the stable cycles are plotted in black. (b) depicts the time series for 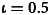 and φ = 0.009. For both figures, the learning and outflow rates are 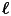 = 1 and *ω* = 0.85.

The cooperation dilemma, unlike the coordination case, can also produce an oscillating dynamical regime. In this case, cooperators may initially establish a high cooperation community, but are unable to maintain it because of the insufficiently steep norm sensitivity curve, akin to how cooperation unravels in repeated game experiments (Fischbacher et al., 2001). That leads to savvy individuals getting disillusioned and leaving at higher rates, making community size come down. However, as the community reverts to mostly naive individuals again, high cooperation can take over again, starting the cycle anew. Figure 8 depicts an example times series of fluctuations present in our model. In Figure 8a, we observe two outcomes: community size either stabilises (represented by the green curves) or fluctuates around that stable value (the black curves). On the other hand, Figure 8b depicts a case where the community grows and crashes in a relatively short period of time. Between these bursts of activity, the community size is minuscule. In this case, there is no stable equilibrium. See Figure SI-2 in Supporting Information-2.2 for the parameter regions that feature these cycles. Such cycling regimes resemble the ‘chasing’ dynamics observed in the laboratory (Ahn, Isaac, & Salmon, 2008; Ehrhart, Keser, et al., 1999; Robbett, 2016), where cooperative groups are undermined by free riders joining and cooperators subsequently moving away. However, in our model the cycling is more closely related to ‘bubbles’ caused by initially optimistic beliefs that subsequently crash before coming back up.

## 4. Discussion

Between the concepts of homo economicus and homo socialis, there is a middle ground where individuals have evolved preferences and acquired social norms that nonetheless are subject to ‘rational’ optimisation with constraints (Akçay, Van Cleve, Feldman, & Roughgarden, 2009; Alger & Weibull, 2013; Fehr & Fischbacher, 2002; Gintis, 2007, 2014; Morsky & Akçay, 2019). Conditional cooperation is an example of this phenomenon. Traxler and Spichtig (2011) showed that conditional cooperation based on norm-dependent relational utilities can sustain cooperation in a community – provided that cooperation is already at a high level. The classic Granovetter–Schelling model also features this tipping-point phenomenon (Granovetter, 1978; Schelling, 1969, 1971). Here, we integrate conditional cooperation norms with a model of community dynamics and show that cooperation can be built from the ground up by judiciously misrepresenting how much people cooperate. Bootstrapped by rosy prospects of prosociality, prosocial communities can emerge and stabilise, overcoming coordination dilemmas and boosting cooperation. Interestingly, this emergence can be quite abrupt, happening in the form of bifurcations that destroy low-cooperation equilibria. These complicated dynamics can explain sudden changes in population behaviour and the rise of communities adopting new social norms.

Our model is analogous to compartmental epidemiological models. Our 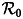, the ratio of entrance to exit rates of the community, plays a fundamental role in determining the presence of polymorphic equilibria of insiders and outsiders as well as whether the community will crash. Social dynamics have frequently been considered through an epidemiological perspective. Although we discuss our model within the framework of cooperation, the model applies to any behaviour on which a norm of positive feedback (i.e. the norm prescribes the behaviour the more it is observed). As such, the model has general implications for norm compliance. Examples include the rapid formation of groups, such as opposition to a government, and how they can be facilitated by social media (McGarty, Thomas, Lala, Smith, & Bliuc, 2014). A further example is boom–bust cycles in the housing market. Busts are less likely to happen after a boom if the optimism is justified as in our coordination dilemma (Burnside, Eichenbaum, & Rebelo, 2016), but repeated booms and bust can happen if optimistic expectations are not fulfilled, as might happen in our cooperation dilemma case. Examples are also found in epidemiology such as conformity to social distancing and vaccination norms, which have been shown to significantly influence the outcomes of epidemiological models (Oraby, Thampi, & Bauch, 2014; Reluga, 2010; Weitz, Park, Eksin, & Dushoff, 2020).

A key aspect of the model is the heterogeneity in how much individuals care about the conditional cooperation norms, as given by the distribution of thresholds for cooperating (Figure 1a). Such heterogeneity has been shown to both hinder and promote cooperation (Chatman & Flynn, 2001; Heckathorn, 1993). Our results show that the effects of such heterogeneity on community dynamics and cooperation level are complicated. On the one hand, heterogeneity can determine whether or not we observe a coordination or cooperation dilemma: low heterogeneity can result in a coordination dilemma where there are both high and low cooperation equilibria, and high heterogeneity can result in a cooperation dilemma with a sole low-cooperation equilibrium (Figure 5a and c). On the other hand, increasing heterogeneity in the coordination dilemma favours cooperation in that it makes the high-cooperation equilibrium globally stable for faster learning (or slower outflow rates; Figure 2b). Yet in cooperation dilemmas, higher heterogeneity moves the high-cooperation equilibrium down, and eventually removes the bifurcation that allows high ratio of outflow to learning rates to switch the community to a high-cooperation equilibrium (Figure 5).

A paradigm in the cooperation literature is that anonymity hinders cooperation and onymity (where partners’ identities and reputations are known) facilitates it (Milinski, Semmann, & Krambeck, 2002; Wang et al., 2017). Onymous settings can promote cooperation by direct or indirect reciprocity (van Apeldoorn & Schram, 2016), partner choice (Smith & Apicella, 2020) or punishment (Lergetporer, Angerer, Glätzle-Rützler, & Sutter, 2014), all of which can materially incentivise cooperation. Indirect reciprocity (Seinen & Schram, 2006; van Apeldoorn & Schram, 2016) as well as partner choice through competitive altruism (Sylwester & Roberts, 2013) have been shown to maintain cooperation in experiments. Our model can be considered as a form of partner choice where individuals choose to leave insufficiently cooperative communities (and thereby ‘associate’ with outsiders, akin to ‘walk away’ models; Aktipis, 2004). Yet the mechanism by which such leaving fosters cooperation is quite different in our model. In partner choice models, leaving effectively penalises uncooperative partners or communities, and selection between individuals or communities promotes cooperation. Here, we do not have selection between communities (because there is only one community). Rather, savvy individuals leaving the community directly increase the amount of cooperation within the community by skewing the remaining community towards naivety, which makes it easier for conditional cooperation to overcome the initial cooperation hurdle.

In this context, direct incentive mechanisms such as punishment can influence the dynamics of conditional cooperators in complex ways. Experimental research has shown that the threat of punishment can increase conditional cooperation (Fehr & Gachter, 2000; Lergetporer et al., 2014). However, punishment has also been shown to increase cheating, which might be due to defectors compensating for being punished by attempting to extract more from the public good (Kirchkamp & Mill, 2020). Players’ knowledge that cheaters can be punished can impact their behaviours. On the one hand, the presence of punishment can signal that free-riding is permissible or prevalent (Bowles & Polania-Reyes, 2012), leading conditional cooperators to expect less cooperation by others and not cooperate themselves. On the other hand, players may believe that punishment will entice others to cooperate, which will in turn induce them to cooperate more via conditional cooperation, as has been shown in children (Lergetporer et al., 2014). Interestingly, in Lergetporer et al. (2014), the children believed that they were more likely to be punished than they actually were, which suggests that more accurate information, perhaps through learning, may reduce cooperation as in our model. In addition, public punishment even in anonymous settings can increase cooperation by informing everyone about the norm or making it more salient (Xiao & Houser, 2011) and increasing its relational utility.

Our model by design does not include downstream incentive consequences of defection through any kind of reciprocity, punishment or partner choice. Instead we focus on how distorted information might interact with conditional cooperation norms. Our results show that paradoxically, under a conditional cooperation norm and in a heterogeneous population, anonymity might foster cooperation if it allows rosy distortions of reality to persist long enough. In an onymous version of our model, players could more quickly identify others and their behaviours, thereby learning the true level of cooperation. This points to a potential trade-off between incentive effects of onymity through mechanisms such as punishment and partner choice and the ability of false beliefs to bootstrap cooperation. An interesting real-world example of this trade-off comes from another online community: the file-sharing software Gnutella introduced a feature that allowed users to only share with other sharers. Users could choose to share only with those who were sharing some threshold number of files or more. While this feature may incentivise cooperation, it also reveals that the sharing norm is not widespread and may constrain growth as new users cannot download content. Our model suggests that counter-intuitively, both latter effects will disfavour a cooperative community. Consistent with this, introducing this feature in Gnutella did not clearly increase cooperation, and may have decreased it (Strahilevitz, 2003).

Our results have interesting implications for strategies to build cooperative communities (or a community following any new norm) in a population of conditional norm followers. We found that the dynamics of community size and cooperation can exhibit hysteresis in important parameters like learning inflow rates: building a cooperative community might require initially high inflow into the community or low learning rates within the community, but once a community is established, it can be maintained at lower inflow or higher learning rates. Likewise, we found that the effect of the outflow rate is non-monotonic; depending on parameters, the community might exist either with low or high outflow rates but not intermediate. Making it hard to leave the community maintains it straightforwardly, but at the expense of increasing the proportion of savvy individuals, which in the cooperation dilemma reduces overall cooperation levels. On the other hand, making it very easy to leave weeds out the savvy individuals, making it possible for high cooperation levels to build up in the community, after which savvy individuals are less likely to leave. Thus, to build a cooperative community, our model prescribes high recruitment effort, relatively opaque learning opportunities and easy outs for savvy individuals.

Our model makes several simplifying assumptions. One significant simplification is the assumption that individuals’ entering and leaving decisions are not correlated with their types. For example, the community could collectively benefit from barriers to entry of individuals with high cooperation thresholds (i.e. individuals that are unlikely to cooperate), which would foster cooperation (Guido, Robbett, & Romaniuc, 2019). One can also consider a bias in outflow from the community, e.g. owing to individuals having lower material or total utilities. If leaving decisions are purely based on material utilities, this would bias the group against cooperation. If leaving is based on total (including relational) utility, the picture becomes more complicated, as those with high thresholds for cooperation can have lower relational utilities. More generally, any bias in joining or leaving that increases the norm sensitivity of the insider population would probably increase the cooperation and the stability of the community. Those that decrease it would probably do the opposite.

Entering and leaving could also be a function of the state of the community: its size or the level of cooperation. In the latter case, the community can experience positive feedback that would reinforce the bistable dynamics. To see this, consider a highly cooperative community under the scenario where influx increases as the cooperation level increases. The high level of cooperation will induce further influx of naive individuals, who are more cooperative than savvy ones, thereby increasing overall cooperation. If, however, cooperation is low, then the influx will be reduced, which in turn further reduces the level of cooperation owing to there being relatively fewer naive individuals.

Another simplifying assumption is that norm sensitivities are distributed normally (or more generally, unimodally). Under this assumption, the basic conditional cooperation model (Section 2.1), may produce one, two or three equilibria. However, one could consider multimodal distributions, which may create several new equilibria beyond the ones considered here if the different modes of the distribution contain high enough fractions of individuals (so that they will intersect with the diagonal in Figure 1a). Traxler (2010) considers multiple equilibria in a threshold model and how belief shocks can impact tax compliance. Although we did not explicitly model multiple equilibria, our model still has something to say about these cases, since the cumulative distribution functions of such distributions will be locally similar to our scenarios between equilibria. In this case, equilibria will still be determined where these curves cross the diagonal, and stability will still be determined by their derivative at those equilibria. As such, we could choose some level of cooperation that the community will broadcast to outsiders, which may be at a stable equilibrium (as in the coordination dilemma) or where the belief is nearly at equilibrium (i.e. at a point close to the diagonal as in the cooperation dilemma of Figure 1a). The scenarios we analyse would thus take place where cooperation is between this naive belief and the first stable equilibrium below it. Considering this multimodal case more generally would be an interesting avenue for future research.

Finally, in this model we take the inaccurate initial beliefs of naive individuals as exogenous. One potential explanation is that inaccurate beliefs might arise from evolved cognitive biases, specifically the well-established bias towards optimism about the future (McKay & Dennett, 2009; Sharot, 2011). Our results suggest that such optimism bias might have an adaptive function in conjunction with conditional cooperation norms, as the rosy view of new communities it produces makes it easier to build and sustain cooperation. Another interesting avenue of research is how such beliefs might form endogenously. It is not difficult to imagine a software company trying to start an active and cooperative community to bias information availability to skew newcomers’ beliefs. However, one might also conjecture that such beliefs emerge endogenously if existing community members recruit others promising higher cooperation. Salience bias (Han, Hirshleifer, & Walden, 2019) can be a consequence of this as cooperation (e.g. making more files available for download) might be more visible than shirking, leading outsiders to overestimate the level of cooperation. Furthermore, recent work by Jackson (2019) shows that in endogenous networks with social interaction, individuals with higher preference for a behaviour form more links with others, leading to higher visibility of such behaviour. In our model, this would mean that individuals with high relational utility for the cooperation norm would be more active in partnering with community members, which would lead to an overestimate of overall cooperation levels. Note that this effect would occur even for savvy individuals, who would have to invest actively to get more representative samples of cooperation to avoid such a bias. That can be a constraint on learning rates in our model. False beliefs can also be due to self-interested misrepresentation by community members, who benefit from an influx of newcomers that cooperate more than the existing community members. The potential origins of biased information about cooperation represents an interesting direction for study.

## Data Availability

The code to run the numerical simulations and make figures is available at https://github.com/bmorsky/communityemergence. For the numerical simulations, we use Julia's DifferentialEquations package (Rackauckas & Nie, 2017).

## Supplementary material

For supplementary material accompanying this paper visit https://doi.org/10.1017/ehs.2021.30. click here to view supplementary material
