# Supplementary material for: False beliefs can bootstrap cooperative communities through social norms
Source: Evol Hum Sci. 2021 Jun 14;3:e36. doi: 10.1017/ehs.2021.30 (PMC10427330; doi:10.1017/ehs.2021.30)
Supplement: Supplementary file 1 [file S2513843X2100030Xsup001.zip › S2513843X2100030Xsup001/SI.pdf]

Supplementary Information for *False beliefs can bootstrap cooperative communities through social norms*

Bryce Morsky* and Erol Akçay†

University of Pennsylvania, Philadelphia, PA, USA

June 7, 2021

SI-1 Conditional cooperation in a public goods game

Here we detail the derivation of the threshold behaviour basing the model off of Traxler and Spichtig (2011) with some minor differences. Individuals within the community have a choice to donate to the public good or not. As such, player i may donate at rate $p_i \in \{0, 1\}$. Those who donate do so at a cost $c > 0$. Players' utilities in the community are composed of a material utility and a relational utility, where the material utility is determined by the proportion of cooperation in the community, $\bar{p} = \sum_{i \in N} s_i / N$, and all costs incurred by the player, and the relational utility is a function of the difference between the expected material utility of the focal player and their belief about the average material utility earned by the other players within the community. Guilt is a potential relational utility. In the case of file-sharing, users were aware of their own upload to download ratio for some applications (Strahilevitz, 2003). As such, they would know how much they themselves were cooperating relative to their perception of the mean level of cooperation. In some applications, the panel showing this could not be hidden.

The material utility for player i in the community is

$$u_i^m(p_i, \bar{p}) = b\bar{p} - cp_i, \tag{SI.1}$$

where $b > 1$ is the benefit. The relational utility, $u_i^r(p_i, q_i)$, is a function of a player's belief of what others are doing, q_i, and the action they take, p_i. We assume that u_i^r is increasing with respect to p_i. Let θ_i be a norm sensitivity parameter for player i (we will assume that $\theta_i \geq 0$, though it could be negative for antisocial individuals). Then, the total expected utility of player i is

$$u_i(p_i, q_i) = bq_i - cp_i + \theta_i u_i^r(p_i, q_i). \tag{SI.2}$$

*morsky@sas.upenn.edu

†eakcay@sas.upenn.edu

A player will donate if $u_i(1, q_i) > u_i(0, q_i)$, i.e.

$$\theta_i > \frac{c}{u_i^r(1, q_i) - u_i^r(0, q_i)}. \tag{SI.3}$$

Thus, player i can increase their utility by cooperating if they believe that a sufficient number of other players are cooperating (i.e. they are a conditional cooperator).

Consider a continuous probability distribution of θ_i with cumulative distribution function $\Phi(\theta)$. Then, the proportion of individuals that cooperate given some q_i is

$$F(q_i) = 1 - \Phi\left(\frac{c}{u_i^r(1, q_i) - u_i^r(0, q_i)}\right). \tag{SI.4}$$

In the main text, we consider F to be the CDF of a normal distribution and that there are two groups with different beliefs, q_i: $\bar{p}$ and $\hat{q}$.

SI-2 Analysis of the model

SI-2.1 Derivation of the model

When interacting with other naive individuals, naive insiders become savvy at rate $\ell(\hat{q}-q)$. And, when interacting with savvy individuals, naive insider become savvy at rate $\ell(\hat{q}-p)$. Averaged across the insider population, the rate is $\ell((\hat{q}-q)(1-y)+(\hat{q}-p)y) = \ell(\hat{q}-\bar{p})$. i.e. the rate at which a naive insider becomes savvy is proportional to the difference between their expectations and reality. The equations for the compartmental model are:

$$\dot{S} = \varphi D - \iota(X+Y)\frac{S}{K}, \tag{SI.5}$$

$$\dot{D} = \omega(\hat{q}-\bar{p})Y - \varphi D, \tag{SI.6}$$

$$\dot{X} = \iota(X+Y)\frac{S}{K} - \ell(\hat{q}-\bar{p})X, \tag{SI.7}$$

$$\dot{Y} = (\hat{q}-\bar{p})(\ell X - \omega Y), \tag{SI.8}$$

where D is the number of discouraged outsiders, X is the number of naive insiders, and Y is the number of savvy insiders. We rewrite our system to be in terms of the number of insiders, $I = X + Y$, and the proportion of savvy insiders, $y = Y/I$. We can determine the dynamics for I as follows:

$$\dot{I} = \dot{X} + \dot{Y} = \iota I\frac{S}{K} - \ell(\hat{q}-\bar{p})X + (\hat{q}-\bar{p})(\ell X - \omega Y) = \iota I\frac{S}{K} - \omega(\hat{q}-\bar{p})yI. \tag{SI.9}$$

And, the dynamics for y can be derived as follows:

$$\begin{aligned}\dot{y} = \frac{\dot{Y}}{I} - \frac{Y\dot{I}}{I^2} &= (\hat{q}-\bar{p})(\ell(1-y) - \omega y) - \iota y\frac{S}{K} + \omega(\hat{q}-\bar{p})y^2 \\ &= (\hat{q}-\bar{p})(1-y)(\ell - \omega y) - \iota y\frac{S}{K}.\end{aligned} \tag{SI.10}$$

Combining these equations with $\dot{S}$ and $\dot{p}$ and substituting in $D = K - S - I$, $I = X + Y$, and $y = Y/I$ gives us:

$$\dot{S} = \varphi(K - S - I) - \iota I \frac{S}{K}, \tag{SI.11}$$

$$\dot{I} = \iota I \frac{S}{K} - \omega(\hat{q} - \bar{p}) y I, \tag{SI.12}$$

$$\dot{y} = (\hat{q} - \bar{p})(1 - y)(\ell - \omega y) - \iota y \frac{S}{K}, \tag{SI.13}$$

$$\dot{p} = F(\bar{p}) - p. \tag{SI.14}$$

SI-2.2 Stability of equilibria

Here we discuss the stability conditions for equilibria. To begin with, consider the boundary equilibria (i.e. cases where $I^* = 0$ or 1) and then the stability of the internal equilibria (i.e. cases where $S* > 0$ and $I^* > 0$).

SI-2.2.1 Stability of all insiders in the coordination dilemma

Theorem 1. *For the coordination game, the high cooperation equilibrium,* $(S^*, I^*, y^*, p^*) = (0, K, y^* \in [0,1], \hat{q})$ *is stable.*

Proof. Consider the Jacobian evaluated at this equilibrium,

$$J^* = \begin{bmatrix} -\varphi & -\varphi - \iota & 0 & 0 \\ \iota & 0 & 0 & \omega K y^{*2} \\ -\iota \frac{y^*}{K} & 0 & 0 & (y^{*2} - y^*)(\ell - \omega y^*) \\ 0 & 0 & 0 & f(\hat{q}) y^* - 1 \end{bmatrix}. \tag{SI.15}$$

The eigenvalues are $-\varphi$, $-\iota$, 0, and $f(\hat{q})y^* - 1 \le f(\hat{q}) - 1 < 0$. But for the zero eigenvalue, these are all negative. Further, the centre eigenspace corresponds to the y-axis. Thus the equilibrium set is stable. □

SI-2.2.2 Stability of the crash equilibrium for the cooperation dilemma

Theorem 2. *If* $\mathcal{R}_0 < 1$ *and* $f(\bar{p}^*) < 1$ *for* y^* *and* p^* *such that* $(\hat{q} - \bar{p}^*)(1 - y^*)(\ell - \omega y^*) - \iota y^* = 0$ *and* $p^* = F(\bar{p}^*)$*, then* $(S^*, I^*, y^*, p^*) = (K, 0, y^*, p^*)$ *is attracting.*

Proof. Though the model is not defined at $(S^*, I^*) = (K, 0)$, we may find y^* as the solution to

$$\begin{aligned} \lim_{S \to K} \dot{y} &= (\hat{q} - \bar{p})(1 - y)(\ell - \omega y) - \iota y \\ &= \omega(q - p)y^3 + (\omega(\hat{q} + p - 2q) - \ell(q - p))y^2 \\ &+ (\ell(2q - p - \hat{q}) - \omega(\hat{q} - q) - \iota)y + \ell(\hat{q} - q) = 0. \end{aligned} \tag{SI.16}$$

Note that by Descartes' rule of signs, if $\omega(\hat{q}+p-2q) > \ell(q-p)$ and $\ell(2q-p-\hat{q}) > \omega(\hat{q}-q)+\iota$, then there is no positive root. With y^* we may find p^*. The Jacobian evaluated at $(K, 0, y^*, p^*)$ is

$$J^* = \begin{bmatrix} -\varphi & -\varphi-\iota & 0 & 0 \\ 0 & \iota-\omega(\hat{q}-\bar{p}^*)y & 0 & 0 \\ -\frac{\iota y^*}{K} & 0 & (q-\hat{q})(\omega-\ell y^*)+(p^*-q)(\omega+(\ell-\omega)y^*)y^* & y^*(y^*-1)(\ell-\omega y^*) \\ 0 & 0 & (p^*-q)f(\bar{p}^*) & y^*f(\bar{p}^*)-1 \end{bmatrix}. \tag{SI.17}$$

And, the characteristic polynomial of J^* is

$$\det(J^* - \lambda I) = \omega(\hat{q}-\bar{p}^*)(\lambda+\varphi)(\lambda+1-\mathcal{R}_0)(\lambda^2-(j_{33}+j_{44})\lambda + j_{33}j_{44} - j_{34}j_{43}), \tag{SI.18}$$

where $j_{ij} \in J$. $-(j_{33}+j_{44}) > 0$, since $j_{33} < 0$ and $j_{44} < 0$. Further, if $f(\bar{p}^*) < 1$, then

$$\begin{aligned} j_{33}j_{44} - j_{34}j_{43} &= \Big((\hat{q}-q)(\omega-\ell y^*) + (q-p^*)(\omega+(\ell-\omega)y^*)y^*\Big)(1-y^*f(\bar{p}^*)) \\ &\quad - y^*(1-y^*)(\ell-\omega y^*)(q-p^*)f(\bar{p}^*) \\ &\geq (q-p^*)\Big((\omega+(\ell-\omega)y^*)y^* - \ell(1-y+y^2)y^*f(\bar{p}^*)\Big) \\ &> (q-p^*)\Big((\omega+(\ell-\omega)y^*)y^* - \ell(1-y+y^2)y^*\Big) \\ &= (q-p^*)y^*(1-y^*)(\omega+\ell y^*) > 0. \end{aligned} \tag{SI.19}$$

Therefore, combined with $\mathcal{R}_0 < 1$, we have that all eigenvalues are negative and thus this state is attracting. □

SI-2.2.3 Stability of internal equilibria

Now consider the internal equilibria (i.e. $I, S \in (0,1)$):

$$S^* = \frac{K}{\mathcal{R}_0}, \tag{SI.20}$$

$$I^* = K\frac{\mathcal{R}_0 - 1}{\mathcal{R}_0 + \iota/\varphi}, \tag{SI.21}$$

$$y^* = \frac{\ell}{\ell+\omega}, \tag{SI.22}$$

$$p^* = F(\bar{p}^*), \tag{SI.23}$$

which exist if $\mathcal{R}_0 > 1$. We begin with a necessary, but not sufficient, theorem for stability, and follow with some numerical results.

Theorem 3. *For an internal equilibrium, if $y^*f(\bar{p}^*) \geq 1$, then the equilibrium cannot be stable.*

Proof. The Jacobian evaluated at these equilibria is

$$J^* = \begin{bmatrix} -\varphi-\iota\frac{I^*}{K} & -\varphi-\iota\frac{S^*}{K} & 0 & 0 \\ \iota\frac{I^*}{K} & 0 & -\omega(\hat{q}-q+2(q-p^*)y^*)I^* & \omega I^* y^{*2} \\ -\iota\frac{y^*}{K} & 0 & -(\hat{q}-q)(\ell+\omega(1-y^*))-(q-p^*)(\ell-2(\ell-\omega)y^*)y^* & -y(1-y)(\ell-\omega y) \\ 0 & 0 & -(q-p^*)f(\bar{p}^*) & y^*f(\bar{p}^*)-1 \end{bmatrix}$$
$$= \begin{bmatrix} -j_1 & -j_2 & 0 & 0 \\ j_3 & 0 & -j_4 & j_5 \\ -j_6 & 0 & -j_7 & -j_8 \\ 0 & 0 & -j_9 & -j_{10} \end{bmatrix}. \tag{SI.24}$$

Here we represent the elements of J^* with $j_i > 0$. Note that for j_7 we have substituted $\iota S^*/K = \omega(\hat{q} - \bar{p}^*)y^*$ to simplify the expression. Stability can be determined by the Routh-Hurwitz criteria. These conditions for our system are:

$$a_3 = j_1 + j_7 + j_{10} > 0, \tag{SI.25}$$
$$a_2 = j_2 j_3 + j_1 j_7 + j_1 j_{10} + j_7 j_{10} - j_8 j_9 > 0, \tag{SI.26}$$
$$a_1 = j_2(j_4 j_6 + j_3(j_7 + j_{10})) + j_1(j_7 j_{10} - j_8 j_9) > 0, \tag{SI.27}$$
$$a_0 = j_2(j_6(j_5 j_9 + j_4 j_{10}) + j_3(j_7 j_{10} - j_8 j_9)) > 0, \tag{SI.28}$$
$$a_1(a_2 a_3 - a_0) - a_0 a_3^2 > 0, \tag{SI.29}$$

where a_i are the coefficients of the characteristic polynomial $P(\lambda) = \sum_0^n a_n \lambda^n$ of J^*. Note that

$$\begin{aligned} a_0 &= j_2\Big((j_5 j_6 - j_3 j_8) j_9 + (j_4 j_6 + j_3 j_7) j_{10}\Big) \\ &= j_2\left((\omega y^{*2} - (1-y^*)(\ell - \omega y^*))\frac{\iota I^* y^* j_9}{K} + (j_4 j_6 + j_3 j_7) j_{10}\right) \\ &= j_2\left(\left(\frac{\ell\omega}{\ell+\omega} - \frac{\ell\omega}{\ell+\omega}\right)\frac{\iota I^* y^* j_9}{K} + (j_4 j_6 + j_3 j_7) j_{10}\right) \\ &= j_2(j_4 j_6 + j_3 j_7)(1 - y^* f(\bar{p}^*)). \end{aligned} \tag{SI.30}$$

If $y^* f(\bar{p}^*) \geq 1$, then $a_0 \leq 0$ and the state cannot be stable. Thus, so long as $y^* f(\bar{p}^*) < 1$, the equilibrium can be stable. Note that this is a necessary, but not sufficient, condition for stability. □

We numerically explored the existence and stability of equilibria across parameter space using the Routh-Hurwitz criteria (Inequalities SI.28-SI.29) and Theorem 2. Figures SI-1 and SI-2 summarize the impact of the parameters, qualitatively, for the coordination and cooperation dilemmas, respectively. For the coordination dilemma, we observe that increasing ℓ can result in the emergence of interior equilibria as well as the system crashing from the single high cooperation equilibrium (Figure SI-1a and SI-1d). ω has the opposite effect; decreasing it can result in the emergence of such equilibria (Figure SI-1b and SI-1d). Notice that for some areas of parameter space, we must traverse the regime with crashing to move from the two non-crashing stable equilibria regime to the sole high cooperation stable equilibrium regime. Varying φ has no effect on the qualitative nature of the system here.

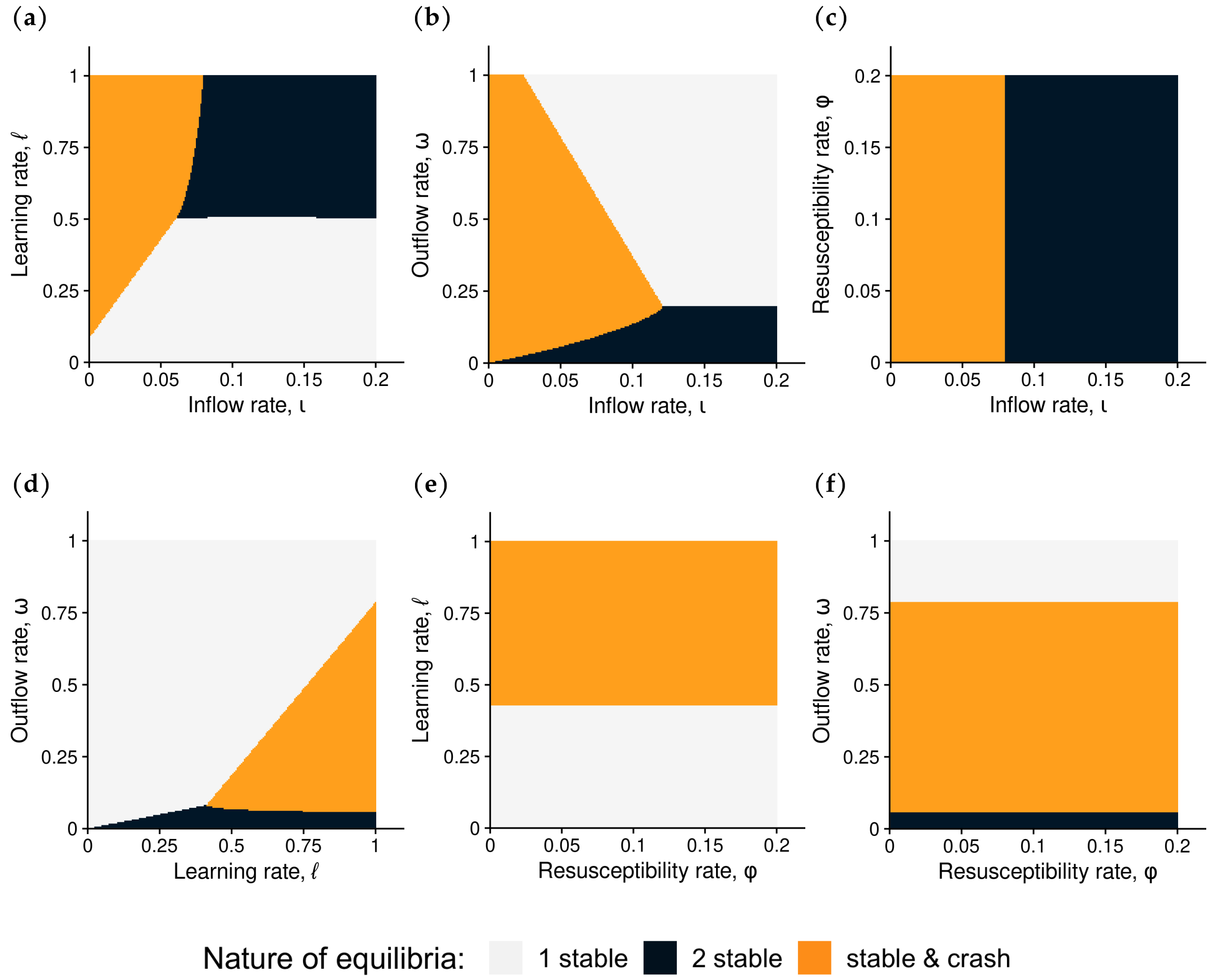

Figure SI-1: Here we plot the qualitative behaviour of the coordination dilemma model across parameter space. We observe the stable high cooperation equilibrium either alone (in grey), with a stable interior point (black), and with a stable crash equilibrium (orange). The default values are $\iota = \varphi = 0.05$, $\ell = 1$, and $\omega = 0.1$. These give us $\omega/\ell = 0.1$, where the two interior equilibria can exist. Stability is determined by the Routh-Hurwitz criteria (Inequalities SI.25-SI.29) and Theorem SI-3.

For the cooperation dilemma, Figure SI-2, we observe a variety of affects of the parameters on the qualitative behaviours the system can have. Increasing ι and decreasing ℓ can pull us from regimes that feature crashing to ones that do not (Figures SI-2a-SI-2c). The intuition behind these results comes from noting that $\mathcal{R}_0$ increases/decreases with respect to ι/ℓ. Varying ι and ℓ can also take us through two other regimes, namely the cycling and two stable interior equilibria regimes. In the cycling regime, we find that no equilibria are stable. We verified through numerical simulations that we observe cycling here. Our figures do not, however, show all regions where cycling can occur. For example, we can have a cycle along with a stable fixed point as shown in Figure 8a of the main manuscript, and thus cycling is possible in the other regimes depicted here.

We discussed the roles of ℓ and ω on the community size in Figure 6 of the main

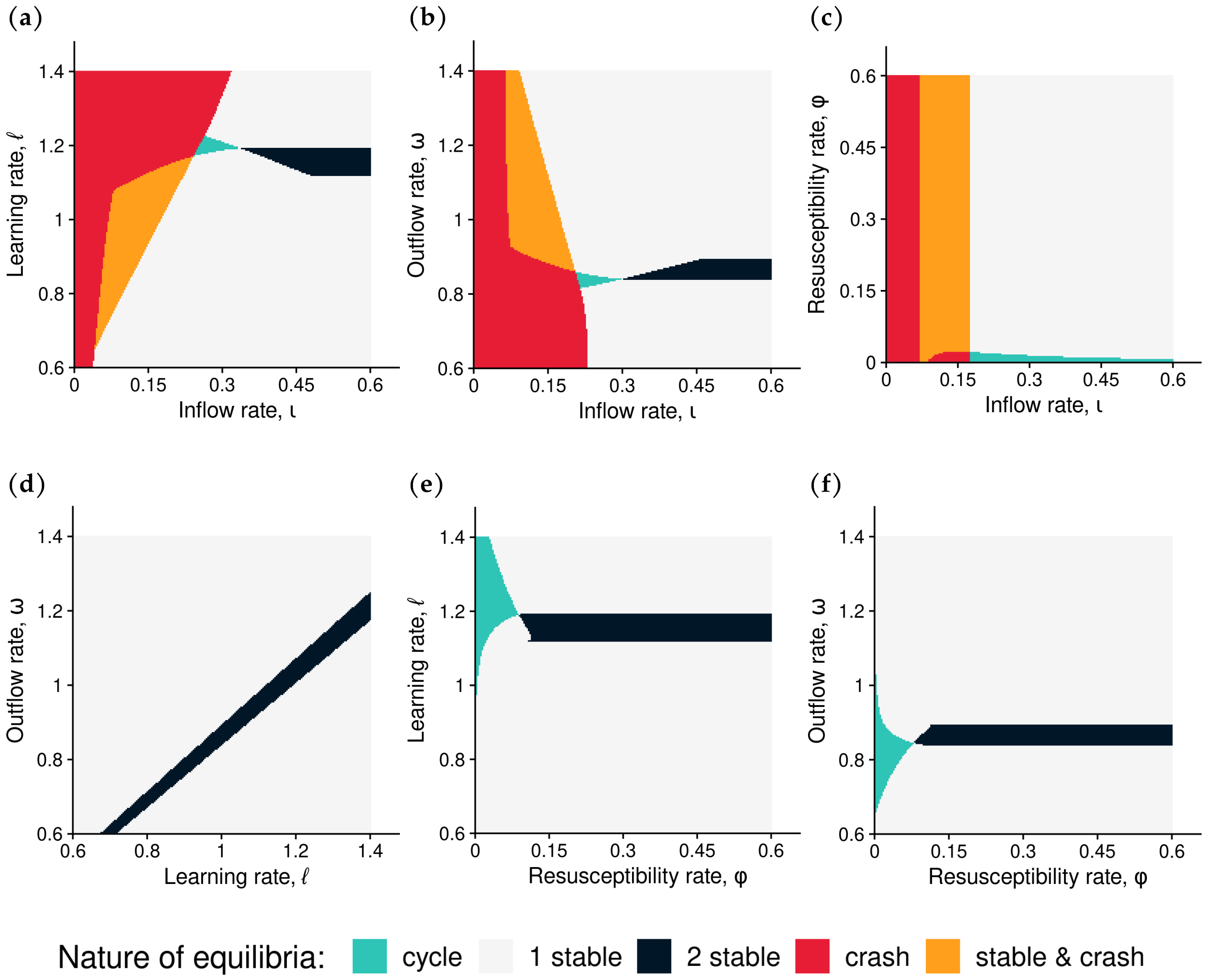

Figure SI-2: Here we plot the qualitative behaviour of the cooperation dilemma model across parameter space. We observe cycling (turquoise), one stable interior equilibrium either (grey), two stable interior equilibria (black), only the crashing equilbirium (red), and stable interior and crashing equilibria (orange). The default values are $\iota = \varphi = 0.5$ and $\ell = \omega = 1$. Stability is determined by the Routh-Hurwitz criteria (Inequalities SI.25-SI.29) and Theorem SI-3.

manuscript. In Figure SI-2d, we see that the two stable equilibria regime is observed for intermediate values of ℓ vs. ω. For higher or lower values of these parameters, we only observe one stable equilibrium. Finally, in Figures SI-2e and SI-2f, we observe that reducing φ can result in cycling when ω/ℓ is low. However, if ω/ℓ is too low, we will have a stable interior equilibrium.

SI-3 Community crashing in the coordination dilemma

Figure SI-3 plots the effects of different parameters on the existence of the crashing state for the coordination dilemma. We observe similar qualitative results to the cooperation dilemma; the community crashes for sufficiently low inflow, high learning, and intermediate outflow rates. Our observation on hysteresis is also applicable here, and more dra-

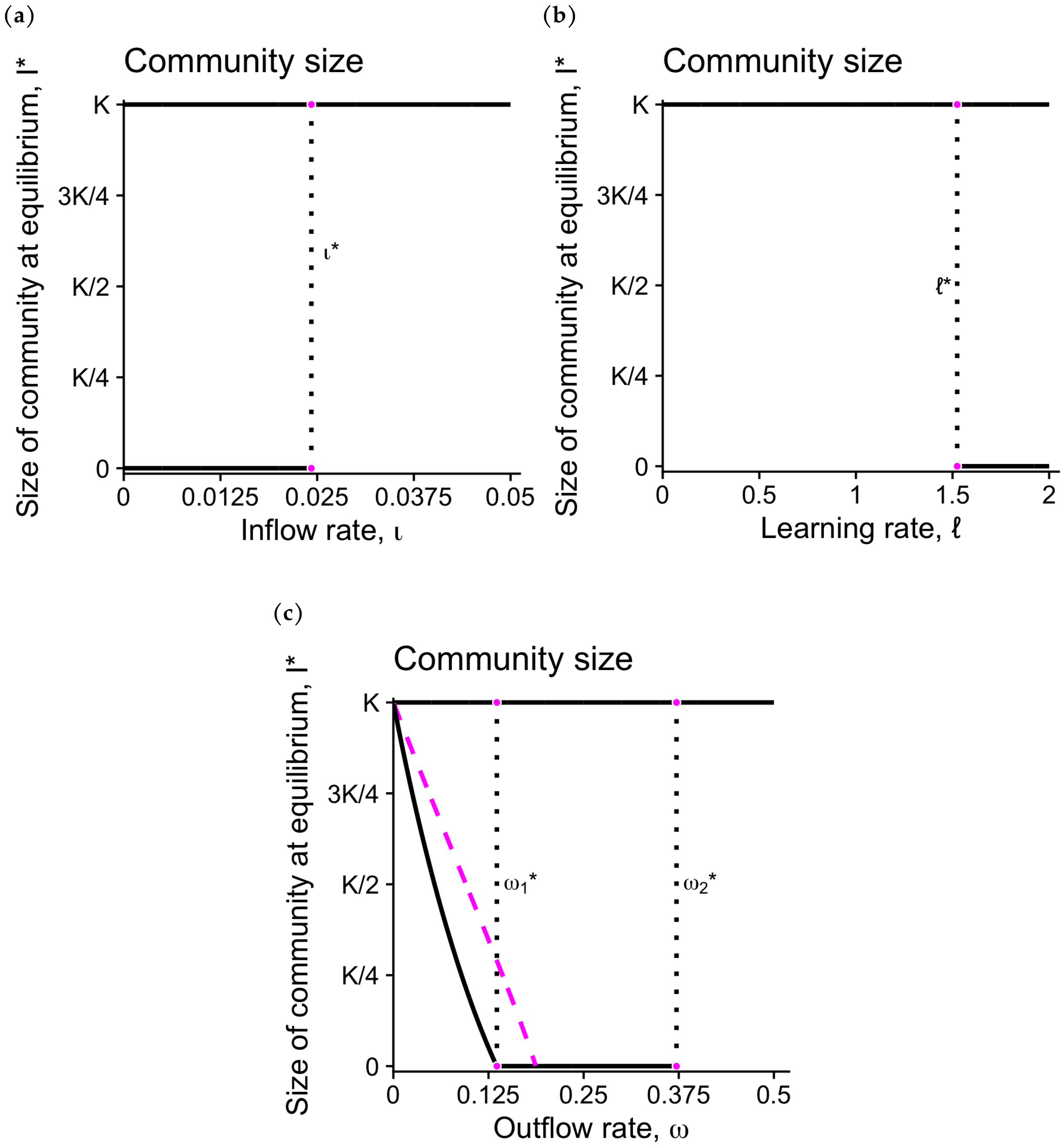

Figure SI-3: The parameters ι, ℓ, and ω (columns 1-3, respectively) determine whether or not the community may crash. (a) Below the threshold ι^*, the community can crash, and above it, it cannot. Here the parameters are $\ell = \omega = \varphi = 1$. (b) Above the threshold ℓ^*, the community can crash, and below it, it cannot. Here the parameters are $\iota = 0.1$ and $\omega = \varphi = 1$. (c) For intermediate ω, between ω_1^* and ω_2^*, the community can crash. Outside of this window, it cannot. Here the parameters are $\iota = 0.1$ and $\ell = \varphi = 1$. The solid black and dashed magenta curves represent the stable and unstable equilibria, respectively, while the dotted lines mark qualitative changes in the system.

matic though less surprising as this is a coordination dilemma. One noticeable difference between the two dilemmas, however, is that community crashing cannot be globally stable here (due to Theorem 1).

References

Strahilevitz, L. J. (2003). Charismatic code, social norms, and the emergence of cooperation on the file-swapping networks. *Virginia Law Review*, 505–595.

Traxler, C., & Spichtig, M. (2011). Social norms and the indirect evolution of conditional cooperation. *Journal of Economics*, *102*(3), 237–262.
